# Supplementary material for: A Phase 2a Randomized, Double-Blind, Dose-Optimizing Study to Evaluate the Immunogenicity and Safety of a Bivalent DNA Vaccine for Hemorrhagic Fever with Renal Syndrome Delivered by Intramuscular Electroporation
Source: Vaccines (Basel). 2020 Jul 11;8(3):377. doi: 10.3390/vaccines8030377 (PMC7565952; doi:10.3390/vaccines8030377)
Supplement: Supplementary file 1 [file vaccines-08-00377-s001.pdf]

Supplemental Data

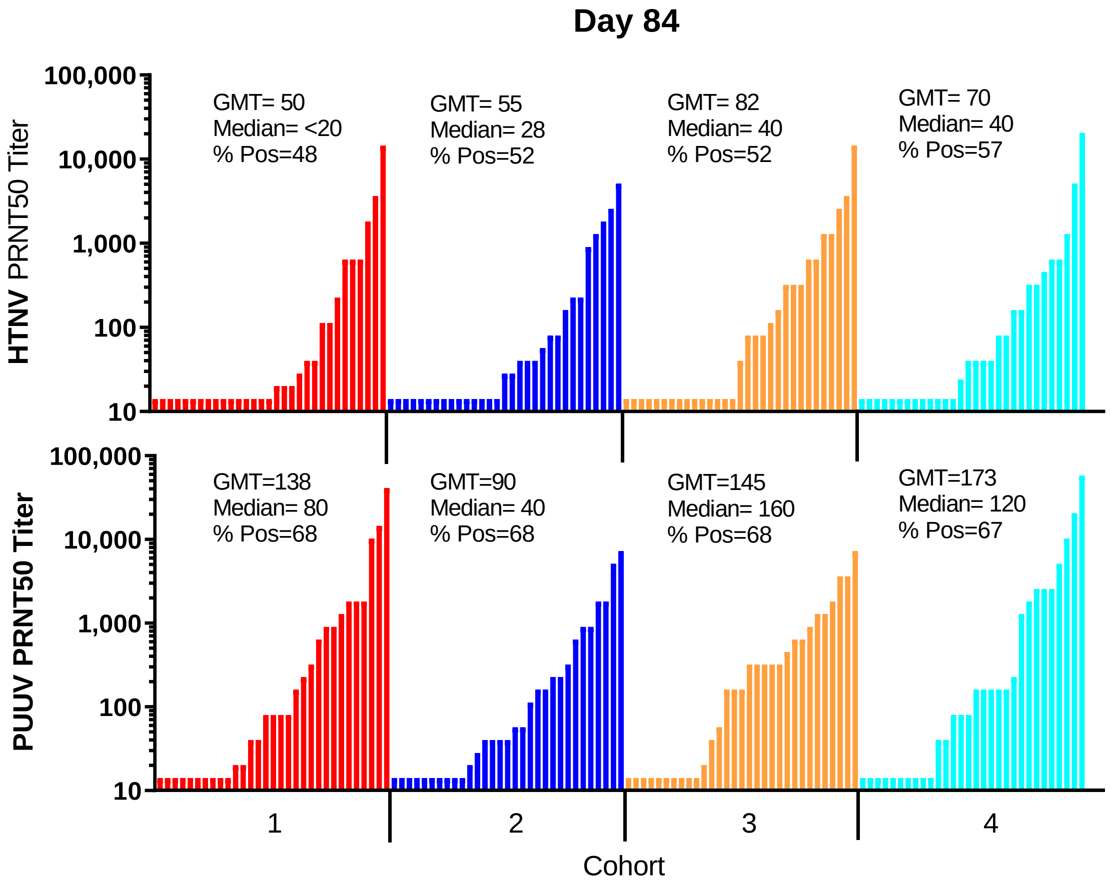

Figure s1. PRNT geometric mean titers (Day 84). Cohort geometric mean titers for Day 84 were determined. Cohorts are the same as defined in Fig 1A. Individual PRNT50 titers were sorted lowest to highest and plotted for HTNV (top panel) and PUUV (bottom panel). GMT, median and % seropositive are plotted.

Table S1. Responders and non-responders in efficacy-evaluable population by sex

Assay	Sex	Total Subjects	Responders Total	% Responders	# Non-Responders	% Non-Responders	p-value
HTNV or PUUV $PsVNA_{50}$	Female	61	55	90.2	6	9.8	0.7931
	Male	62	55	88.7	7	11.3	.
HTNV or PUUV $PsVNA_{80}$	Female	61	49	80.3	12	19.7	0.7837
	Male	62	51	82.3	11	17.7	.
HTNV $PsVNA_{50}$	Female	61	50	82.0	11	18.0	0.4313
	Male	62	54	87.1	8	12.9	.
HTNV $PsVNA_{80}$	Female	61	40	65.6	21	34.4	0.1455
	Male	62	48	77.4	14	22.6	.
PUUV $PsVNA_{50}$	Female	61	50	82.0	11	18.0	0.5309
	Male	62	48	77.4	14	22.6	.
PUUV $PsVNA_{80}$	Female	61	40	65.6	21	34.4	0.6545
	Male	62	43	69.4	19	30.6	.

Note: p-values
are from Chi-Squared tests

Table S2. Responders and non-responders in efficacy-evaluable population by race

Assay	Category	Total Subjects	Responders #	Responders %	Non-Responders #	Non-Responders %	p-value
HTNV or PUUV $PsVNA_{50}$	American Indian/ Alaskan Native	2	2	100.0	0	0.0	0.8690
	Asian	4	4	100.0	0	0.0	.
	Black/ African American	61	53	86.9	8	13.1	.
	American Indian/ Alaskan Native	1	1	100.0	0	0.0	.
	Other	5	5	100.0	0	0.0	.
	White	50	45	90.0	5	10.0	.
HTNV or PUUV $PsVNA_{80}$	American Indian/ Alaskan Native	2	2	100.0	0	0.0	0.7643
	Asian	4	4	100.0	0	0.0	.
	Black/ African American	61	47	77.0	14	23.0	.
	Native Hawaiian/ Other Pacific Islander	1	1	100.0	0	0.0	.
	Other	5	4	80.0	1	20.0	.
	White	50	42	84.0	8	16.0	.
HTNV $PsVNA_{50}$	American Indian/ Alaskan Native	2	2	100.0	0	0.0	0.8825
	Asian	4	4	100.0	0	0.0	.
	Black/ African American	61	50	82.0	11	18.0	.
	Native Hawaiian/ Other Pacific Islander	1	1	100.0	0	0.0	.
	Other	5	4	80.0	1	20.0	.
	White	50	43	86.0	7	14.0	.
HTNV $PsVNA_{80}$	American Indian/ Alaskan Native	2	2	100.0	0	0.0	0.6687
	Asian	4	4	100.0	0	0.0	.

Table S3. Responders and non-responders in efficacy-evaluable population by ethnicity

Assay	Category	Total Subjects	Responders #	Responders %	Non-Responders #	Non-Responders %	p-value
HTNV or PUUV PsVNA$_{50}$	Hispanic/Latino	11	10	90.9	1	9.1	0.8673
	Not Hispanic/Latino	112	100	89.3	12	10.7	.
HTNV or PUUV PsVNA$_{80}$	Hispanic/Latino	11	8	72.7	3	27.3	0.4447
	Not Hispanic/Latino	112	92	82.1	20	17.9	.
HTNV PsVNA$_{50}$	Hispanic/Latino	11	9	81.8	2	18.2	0.7926
	Not Hispanic/Latino	112	95	84.8	17	15.2	.
HTNV PsVNA$_{80}$	Hispanic/Latino	11	6	54.5	5	45.5	0.1904
	Not Hispanic/Latino	112	82	73.2	30	26.8	.
PUUV PsVNA$_{50}$	Hispanic/Latino	11	10	90.9	1	9.1	0.3319
	Not Hispanic/Latino	112	88	78.6	24	21.4	.
PUUV PsVNA$_{80}$	Hispanic/Latino	11	8	72.7	3	27.3	0.6970
	Not Hispanic/Latino	112	75	67.0	37	33.0	.
PsVNA$_{80}$	Alaskan Native	2	1	50.0	1	50.0	0.7781
	Asian	4	2	50.0	2	50.0	.
	Black/ African American	61	39	63.9	22	36.1	.
	Native Hawaiian/ Other Pacific Islander	1	1	100.0	0	0.0	.
	Other	5	4	80.0	1	20.0	.
	White	50	36	72.0	14	28.0	.

Note: p-values are from Chi-Squared tests

Note: p-values are from Chi-Squared tests

Table S4. Responders and non-responders in efficacy-evaluable population by age

**Assay**	**Total subjects**	**Responders #**	**Responder Mean age (years)**	**Non-responders #**	**Non-responder mean age (years)**	**p-value**
HTNV or PUUV $PsVNA_{50}$	123	110	32.1	13	33.0	0.6446
HTNV or PUUV $PsVNA_{80}$	123	100	31.6	23	34.8	0.1531
HTNV $PsVNA_{50}$	123	104	32.0	19	33.3	0.4513
HTNV $PsVNA_{80}$	123	88	31.9	35	32.8	0.8202
PUUV $PsVNA_{50}$	123	98	31.7	25	33.9	0.2977
PUUV $PsVNA_{80}$	123	83	31.1	40	34.5	**0.0504**

Note: p-values are from two-sided Wilcoxon tests

Table S5. Association of HLA Allele Targets and PUUV PsVNA50 Titer Response

Allele Target	Allele Status	Responder n (%)	Non-Responder n (%)	Odds Ratio (95% Confidence Limit)	Unadjusted p value	False Discovery Rate Adjusted p value
A*02	Target Present	34 (14.3%)	14 (5.9%)	0.952 (0.463, 1.960)	0.8941	0.9889
A*02	Other Present	102 (42.9%)	40 (16.8%)		.	.
A*02	Absent	36 (15.1%)	12 (5.0%)		.	.
A*03	Target Present	13 (5.5%)	7 (2.9%)	0.710 (0.267, 1.888)	0.4919	0.7561
A*03	Other Present	123 (51.7%)	47 (19.7%)		.	.
A*03	Absent	36 (15.1%)	12 (5.0%)		.	.
B*15	Target Present	13 (5.5%)	10 (4.2%)	0.416 (0.169, 1.021)	0.0556	0.4447
B*15	Other Present	125 (52.5%)	40 (16.8%)		.	.
B*15	Absent	34 (14.3%)	16 (6.7%)		.	.
B*35	Target Present	17 (7.1%)	3 (1.3%)	2.201 (0.616, 7.859)	0.2244	0.7405
B*35	Other Present	121 (50.8%)	47 (19.7%)		.	.
B*35	Absent	34 (14.3%)	16 (6.7%)		.	.
C*02	Target Present	18 (7.6%)	8 (3.4%)	1.007 (0.399, 2.541)	0.9889	0.9889
C*02	Other Present	76 (31.9%)	34 (14.3%)		.	.
C*02	Absent	78 (32.8%)	24 (10.1%)		.	.
C*03	Target Present	18 (7.6%)	8 (3.4%)	1.007 (0.399, 2.541)	0.9889	0.9889
C*03	Other Present	76 (31.9%)	34 (14.3%)		.	.
C*03	Absent	78 (32.8%)	24 (10.1%)		.	.
C*04	Target Present	13 (5.5%)	4 (1.7%)	1.524 (0.466, 4.986)	0.4856	0.7561
C*04	Other Present	81 (34.0%)	38 (16.0%)		.	.
C*04	Absent	78 (32.8%)	24 (10.1%)		.	.
C*07	Target Present	10 (4.2%)	7 (2.9%)	0.595 (0.210, 1.689)	0.3296	0.7561
C*07	Other Present	84 (35.3%)	35 (14.7%)		.	.
C*07	Absent	78 (32.8%)	24 (10.1%)		.	.
DPB1*01	Target Present	14 (5.9%)	7 (2.9%)	0.661 (0.250, 1.750)	0.4049	0.7561
DPB1*01	Other Present	124 (52.1%)	41 (17.2%)		.	.
DPB1*01	Absent	34 (14.3%)	18 (7.6%)		.	.
DQB1*02	Target Present	33 (13.9%)	12 (5.0%)	1.047 (0.493, 2.227)	0.9043	0.9889
DQB1*02	Other Present	105 (44.1%)	40 (16.8%)		.	.
DQB1*02	Absent	34 (14.3%)	14 (5.9%)		.	.
DQB1*03	Target Present	39 (16.4%)	15 (6.3%)	0.972 (0.480, 1.967)	0.9363	0.9889
DQB1*03	Other Present	99 (41.6%)	37 (15.5%)		.	.
DQB1*03	Absent	34 (14.3%)	14 (5.9%)		.	.
DQB1*05	Target Present	18 (7.6%)	9 (3.8%)	0.717 (0.299, 1.715)	0.4543	0.7561

Allele Target	Allele Status	Responder n (%)	Non-Responder n (%)	Odds Ratio (95% Confidence Limit)	Unadjusted p value	False Discovery Rate Adjusted p value
DQB1*05	Other Present	120 (50.4%)	43 (18.1%)		.	.
DQB1*05	Absent	34 (14.3%)	14 (5.9%)		.	.
DQB1*06	Target Present	45 (18.9%)	11 (4.6%)	1.804 (0.848, 3.836)	0.1256	0.6701
DQB1*06	Other Present	93 (39.1%)	41 (17.2%)		.	.
DQB1*06	Absent	34 (14.3%)	14 (5.9%)		.	.
DRA*01:01	Target Present	20 (8.4%)	9 (3.8%)	0.818 (0.343, 1.948)	0.6494	0.8479
DRA*01:01	Other Present	106 (44.5%)	39 (16.4%)		.	.
DRA*01:01	Absent	46 (19.3%)	18 (7.6%)		.	.
DRA*01:02	Target Present	13 (5.5%)	8 (3.4%)	0.575 (0.222, 1.489)	0.2545	0.7405
DRA*01:02	Other Present	113 (47.5%)	40 (16.8%)		.	.
DRA*01:02	Absent	46 (19.3%)	18 (7.6%)		.	.
DRA*02:01	Target Present	47 (19.7%)	15 (6.3%)	1.309 (0.644, 2.660)	0.4569	0.7561
DRA*02:01	Other Present	79 (33.2%)	33 (13.9%)		.	.
DRA*02:01	Absent	46 (19.3%)	18 (7.6%)		.	.
DRA*02:02	Target Present	33 (13.9%)	15 (6.3%)	0.781 (0.377, 1.617)	0.5050	0.7561
DRA*02:02	Other Present	93 (39.1%)	33 (13.9%)		.	.
DRA*02:02	Absent	46 (19.3%)	18 (7.6%)		.	.
DRA*02:03	Target Present	13 (5.5%)	1 (0.4%)	5.407 (0.688, 42.516)	0.1087	0.6701
DRA*02:03	Other Present	113 (47.5%)	47 (19.7%)		.	.
DRA*02:03	Absent	46 (19.3%)	18 (7.6%)		.	.
DRB1*03	Target Present	15 (6.3%)	13 (5.5%)	0.367 (0.161, 0.834)	**0.0167**	0.4447
DRB1*03	Other Present	129 (54.2%)	41 (17.2%)		.	.
DRB1*03	Absent	28 (11.8%)	12 (5.0%)		.	.
DRB1*04	Target Present	20 (8.4%)	5 (2.1%)	1.580 (0.562, 4.444)	0.3860	0.7561
DRB1*04	Other Present	124 (52.1%)	49 (20.6%)		.	.
DRB1*04	Absent	28 (11.8%)	12 (5.0%)		.	.
DRB1*07	Target Present	20 (8.4%)	5 (2.1%)	1.580 (0.562, 4.444)	0.3860	0.7561
DRB1*07	Other Present	124 (52.1%)	49 (20.6%)		.	.
DRB1*07	Absent	28 (11.8%)	12 (5.0%)		.	.
DRB1*13	Target Present	36 (15.1%)	6 (2.5%)	2.667 (1.053, 6.750)	**0.0385**	0.4447
DRB1*13	Other Present	108 (45.4%)	48 (20.2%)		.	.
DRB1*13	Absent	28 (11.8%)	12 (5.0%)		.	.
DRB1*15	Target Present	22 (9.2%)	6 (2.5%)	1.442 (0.551, 3.776)	0.4557	0.7561
DRB1*15	Other Present	122 (51.3%)	48 (20.2%)		.	.
DRB1*15	Absent	28 (11.8%)	12 (5.0%)		.	.

Allele Target	Allele Status	Responder n (%)	Non-Responder n (%)	Odds Ratio (95% Confidence Limit)	Unadjusted p value	False Discovery Rate Adjusted p value
DRB3*01	Target Present	34 (14.3%)	13 (5.5%)	0.902 (0.399, 2.041)	0.8042	0.9889
DRB3*01	Other Present	58 (24.4%)	20 (8.4%)		.	.
DRB3*01	Absent	80 (33.6%)	33 (13.9%)		.	.
DRB3*02	Target Present	35 (14.7%)	13 (5.5%)	0.945 (0.418, 2.135)	0.8912	0.9889
DRB3*02	Other Present	57 (23.9%)	20 (8.4%)		.	.
DRB3*02	Absent	80 (33.6%)	33 (13.9%)		.	.
DRB3*03	Target Present	23 (9.7%)	7 (2.9%)	1.238 (0.475, 3.229)	0.6624	0.8479
DRB3*03	Other Present	69 (29.0%)	26 (10.9%)		.	.
DRB3*03	Absent	80 (33.6%)	33 (13.9%)		.	.
G*01:01	Target Present	78 (32.8%)	22 (9.2%)	2.127 (1.003, 4.512)	**0.0491**	0.4447
G*01:01	Other Present	30 (12.6%)	18 (7.6%)		.	.
G*01:01	Absent	64 (26.9%)	26 (10.9%)		.	.
G*01:04	Target Present	20 (8.4%)	11 (4.6%)	0.599 (0.257, 1.397)	0.2358	0.7405
G*01:04	Other Present	88 (37.0%)	29 (12.2%)		.	.
G*01:04	Absent	64 (26.9%)	26 (10.9%)		.	.
H*01	Target Present	46 (19.3%)	16 (6.7%)	1.265 (0.593, 2.700)	0.5435	0.7561
H*01	Other Present	50 (21.0%)	22 (9.2%)		.	.
H*01	Absent	76 (31.9%)	28 (11.8%)		.	.
H*02	Target Present	50 (21.0%)	22 (9.2%)	0.791 (0.370, 1.687)	0.5435	0.7561
H*02	Other Present	46 (19.3%)	16 (6.7%)		.	.
H*02	Absent	76 (31.9%)	28 (11.8%)		.	.
J*01	Target Present	72 (30.3%)	26 (10.9%)	0.503 (0.158, 1.600)	0.2446	0.7405
J*01	Other Present	22 (9.2%)	4 (1.7%)		.	.
J*01	Absent	78 (32.8%)	36 (15.1%)		.	.
J*02	Target Present	22 (9.2%)	4 (1.7%)	1.986 (0.625, 6.310)	0.2446	0.7405
J*02	Other Present	72 (30.3%)	26 (10.9%)		.	.
J*02	Absent	78 (32.8%)	36 (15.1%)		.	.

Responder defined as PsVNA50 titer > 20. P value determined by logistic regression.

Table S6. Association of HLA Allele Targets and HTNV PsVNA50 Titer Response

Allele Target	Allele Status	Responder n (%)	Non-Responder n (%)	Odds Ratio (95% Confidence Limit)	Unadjusted p value	False Discovery Rate Adjusted p value
A*02	Target Present	30 (12.6%)	18 (7.6%)	0.446 (0.220, 0.908)	**0.0260**	0.4047
A*02	Other Present	112 (47.1%)	30 (12.6%)		.	.
A*02	Absent	42 (17.6%)	6 (2.5%)		.	.
A*03	Target Present	15 (6.3%)	5 (2.1%)	1.016 (0.349, 2.960)	0.9772	0.9772
A*03	Other Present	127 (53.4%)	43 (18.1%)		.	.
A*03	Absent	42 (17.6%)	6 (2.5%)		.	.
B*15	Target Present	12 (5.0%)	11 (4.6%)	0.294 (0.120, 0.722)	**0.0076**	0.2425
B*15	Other Present	130 (54.6%)	35 (14.7%)		.	.
B*15	Absent	42 (17.6%)	8 (3.4%)		.	.
B*35	Target Present	16 (6.7%)	4 (1.7%)	1.333 (0.422, 4.210)	0.6240	0.9772
B*35	Other Present	126 (52.9%)	42 (17.6%)		.	.
B*35	Absent	42 (17.6%)	8 (3.4%)		.	.
C*02	Target Present	18 (7.6%)	8 (3.4%)	0.844 (0.332, 2.144)	0.7210	0.9772
C*02	Other Present	80 (33.6%)	30 (12.6%)		.	.
C*02	Absent	86 (36.1%)	16 (6.7%)		.	.
C*03	Target Present	17 (7.1%)	9 (3.8%)	0.676 (0.272, 1.684)	0.4007	0.8015
C*03	Other Present	81 (34.0%)	29 (12.2%)		.	.
C*03	Absent	86 (36.1%)	16 (6.7%)		.	.
C*04	Target Present	12 (5.0%)	5 (2.1%)	0.921 (0.301, 2.816)	0.8852	0.9772
C*04	Other Present	86 (36.1%)	33 (13.9%)		.	.
C*04	Absent	86 (36.1%)	16 (6.7%)		.	.
C*07	Target Present	15 (6.3%)	2 (0.8%)	3.252 (0.707, 14.964)	0.1299	0.5196
C*07	Other Present	83 (34.9%)	36 (15.1%)		.	.
C*07	Absent	86 (36.1%)	16 (6.7%)		.	.
DPB1*01	Target Present	15 (6.3%)	6 (2.5%)	0.800 (0.291, 2.200)	0.6654	0.9772
DPB1*01	Other Present	125 (52.5%)	40 (16.8%)		.	.
DPB1*01	Absent	44 (18.5%)	8 (3.4%)		.	.
DQB1*02	Target Present	35 (14.7%)	10 (4.2%)	1.243 (0.562, 2.751)	0.5915	0.9772
DQB1*02	Other Present	107 (45.0%)	38 (16.0%)		.	.
DQB1*02	Absent	42 (17.6%)	6 (2.5%)		.	.
DQB1*03	Target Present	36 (15.1%)	18 (7.6%)	0.566 (0.282, 1.135)	0.1089	0.5196
DQB1*03	Other Present	106 (44.5%)	30 (12.6%)		.	.
DQB1*03	Absent	42 (17.6%)	6 (2.5%)		.	.
DQB1*05	Target Present	21 (8.8%)	6 (2.5%)	1.215 (0.459, 3.214)	0.6950	0.9772

Allele Target	Allele Status	Responder n (%)	Non-Responder n (%)	Odds Ratio (95% Confidence Limit)	Unadjusted p value	False Discovery Rate Adjusted p value
DQB1*05	Other Present	121 (50.8%)	42 (17.6%)		.	.
DQB1*05	Absent	42 (17.6%)	6 (2.5%)		.	.
DQB1*06	Target Present	47 (19.7%)	9 (3.8%)	2.144 (0.959, 4.794)	0.0632	0.4047
DQB1*06	Other Present	95 (39.9%)	39 (16.4%)		.	.
DQB1*06	Absent	42 (17.6%)	6 (2.5%)		.	.
DRA*01:01	Target Present	18 (7.6%)	11 (4.6%)	0.521 (0.225, 1.207)	0.1283	0.5196
DRA*01:01	Other Present	110 (46.2%)	35 (14.7%)		.	.
DRA*01:01	Absent	56 (23.5%)	8 (3.4%)		.	.
DRA*01:02	Target Present	15 (6.3%)	6 (2.5%)	0.885 (0.321, 2.437)	0.8131	0.9772
DRA*01:02	Other Present	113 (47.5%)	40 (16.8%)		.	.
DRA*01:02	Absent	56 (23.5%)	8 (3.4%)		.	.
DRA*02:01	Target Present	47 (19.7%)	15 (6.3%)	1.199 (0.587, 2.448)	0.6179	0.9772
DRA*02:01	Other Present	81 (34.0%)	31 (13.0%)		.	.
DRA*02:01	Absent	56 (23.5%)	8 (3.4%)		.	.
DRA*02:02	Target Present	36 (15.1%)	12 (5.0%)	1.109 (0.517, 2.377)	0.7909	0.9772
DRA*02:02	Other Present	92 (38.7%)	34 (14.3%)		.	.
DRA*02:02	Absent	56 (23.5%)	8 (3.4%)		.	.
DRA*02:03	Target Present	12 (5.0%)	2 (0.8%)	2.276 (0.490, 10.580)	0.2942	0.7053
DRA*02:03	Other Present	116 (48.7%)	44 (18.5%)		.	.
DRA*02:03	Absent	56 (23.5%)	8 (3.4%)		.	.
DRB1*03	Target Present	17 (7.1%)	11 (4.6%)	0.430 (0.185, 0.997)	**0.0493**	0.4047
DRB1*03	Other Present	133 (55.9%)	37 (15.5%)		.	.
DRB1*03	Absent	34 (14.3%)	6 (2.5%)		.	.
DRB1*04	Target Present	16 (6.7%)	9 (3.8%)	0.517 (0.212, 1.261)	0.1473	0.5236
DRB1*04	Other Present	134 (56.3%)	39 (16.4%)		.	.
DRB1*04	Absent	34 (14.3%)	6 (2.5%)		.	.
DRB1*07	Target Present	23 (9.7%)	2 (0.8%)	4.165 (0.945, 18.367)	0.0595	0.4047
DRB1*07	Other Present	127 (53.4%)	46 (19.3%)		.	.
DRB1*07	Absent	34 (14.3%)	6 (2.5%)		.	.
DRB1*13	Target Present	35 (14.7%)	7 (2.9%)	1.783 (0.735, 4.325)	0.2012	0.5364
DRB1*13	Other Present	115 (48.3%)	41 (17.2%)		.	.
DRB1*13	Absent	34 (14.3%)	6 (2.5%)		.	.
DRB1*15	Target Present	24 (10.1%)	4 (1.7%)	2.095 (0.689, 6.375)	0.1926	0.5364
DRB1*15	Other Present	126 (52.9%)	44 (18.5%)		.	.
DRB1*15	Absent	34 (14.3%)	6 (2.5%)		.	.

Allele Target	Allele Status	Responder n (%)	Non-Responder n (%)	Odds Ratio (95% Confidence Limit)	Unadjusted p value	False Discovery Rate Adjusted p value
DRB3*01	Target Present	35 (14.7%)	12 (5.0%)	0.939 (0.408, 2.165)	0.8831	0.9772
DRB3*01	Other Present	59 (24.8%)	19 (8.0%)		.	.
DRB3*01	Absent	90 (37.8%)	23 (9.7%)		.	.
DRB3*02	Target Present	36 (15.1%)	12 (5.0%)	0.983 (0.427, 2.262)	0.9674	0.9772
DRB3*02	Other Present	58 (24.4%)	19 (8.0%)		.	.
DRB3*02	Absent	90 (37.8%)	23 (9.7%)		.	.
DRB3*03	Target Present	23 (9.7%)	7 (2.9%)	1.111 (0.423, 2.913)	0.8311	0.9772
DRB3*03	Other Present	71 (29.8%)	24 (10.1%)		.	.
DRB3*03	Absent	90 (37.8%)	23 (9.7%)		.	.
G*01:01	Target Present	75 (31.5%)	25 (10.5%)	1.645 (0.781, 3.465)	0.1901	0.5364
G*01:01	Other Present	31 (13.0%)	17 (7.1%)		.	.
G*01:01	Absent	78 (32.8%)	12 (5.0%)		.	.
G*01:04	Target Present	21 (8.8%)	10 (4.2%)	0.791 (0.336, 1.860)	0.5904	0.9772
G*01:04	Other Present	85 (35.7%)	32 (13.4%)		.	.
G*01:04	Absent	78 (32.8%)	12 (5.0%)		.	.
H*01	Target Present	39 (16.4%)	23 (9.7%)	0.698 (0.339, 1.440)	0.3306	0.7053
H*01	Other Present	51 (21.4%)	21 (8.8%)		.	.
H*01	Absent	94 (39.5%)	10 (4.2%)		.	.
H*02	Target Present	51 (21.4%)	21 (8.8%)	1.432 (0.695, 2.953)	0.3306	0.7053
H*02	Other Present	39 (16.4%)	23 (9.7%)		.	.
H*02	Absent	94 (39.5%)	10 (4.2%)		.	.
J*01	Target Present	71 (29.8%)	27 (11.3%)	0.969 (0.366, 2.564)	0.9493	0.9772
J*01	Other Present	19 (8.0%)	7 (2.9%)		.	.
J*01	Absent	94 (39.5%)	20 (8.4%)		.	.
J*02	Target Present	19 (8.0%)	7 (2.9%)	1.032 (0.390, 2.731)	0.9493	0.9772
J*02	Other Present	71 (29.8%)	27 (11.3%)		.	.
J*02	Absent	94 (39.5%)	20 (8.4%)		.	.

Responder defined as PsVNA50 titer > 20. P value determined by logistic regression.
